# Abdominal tuberculosis misdiagnosed as acute surgical abdomen and carcinomatosis

**DOI:** 10.12688/f1000research.53036.1

**Published:** 2021-05-07

**Authors:** Edinson Dante Meregildo-Rodriguez, Rosita Claudia Tafur-Ramirez, Walter Giovanny Espino-Saavedra, Sonia Fiorella Angulo-Prentice

**Affiliations:** 1 Universidad César Vallejo, Escuela de Medicina, Trujillo, La Libertad, Peru; 2 Department of Internal Medicine, Hospital Regional Lambayeque, Chiclayo, Lambayeque, 14007, Peru; 3 Department of Nephrology, Hospital Regional Lambayeque, Chiclayo, Lambayeque, 14007, Peru; 4 Department of Clinical Pathology, Hospital Regional Lambayeque, Chiclayo, Lambayeque, 14007, Peru

**Keywords:** acute abdomen, gastrointestinal tuberculosis, tuberculosis, tuberculous peritonitis, neoplasm, cancer

## Abstract

Tuberculosis is a major public health problem worldwide. Tuberculosis can be confused with other diseases and its diagnosis is frequently delayed, especially in areas of low prevalence. We report two cases of young patients who presented preoperatively as acute abdomen due to acute appendicitis. During the intra-operatory these cases were misdiagnosed as “carcinomatosis”, and in the postoperative period these cases were complicated with septic shock. In both cases, histopathology showed caseating granulomas which suggested tuberculous peritonitis and enteritis. Subsequently, RT-PCR in peritoneal fluid confirmed *Mycobacterium tuberculosis*. In one case the clinical response to treatment was excellent, and the other case was fatal.

The aim of this report is to bring attention to the spectrum of tuberculosis, and to serve as a reminder of tuberculosis as the great imitator that can masquerade as cancer. Most tuberculous patients erroneously diagnosed as cancer have extensive “neoplastic” lesions that would suggest an advanced-stage malignancy. Assuming a case as an advanced cancer would reduce the chance of performing more exhaustive studies to get a definitive diagnosis and clinicians would be tempted to offer only palliative treatments.

## Introduction

Tuberculosis (TB) is the most widespread infectious disease and one of the leading causes of death worldwide. ^1- 3^ The proportion of patients with extra-pulmonary tuberculosis has risen during the last decades all over the world. ^1, 2^ Tuberculosis in Peru ranks fifteenth among the causes of death, and predominantly affects the poorest social strata. ^2^ Abdominal tuberculosis is defined as infection of the peritoneum, omentum, mesentery, lymphatics nodes, and hollow or solid abdominal organs such as the liver, spleen, and pancreas with *Mycobacterium tuberculosis*. ^4- 7^ Abdominal TB accounts for about 5% of all TB cases and 15% of extrapulmonary TB forms and is an increasingly common disease that can have bizarre presentations. ^8- 11^

## Case reports

Herein, we present two cases of abdominal TB mimicking at the presentation an acute abdomen related to surgical aspect of appendicitis, “carcinomatosis”, and septic shock. This report aims to suggest to clinicians the need of including TB in the differential diagnosis of pulmonary and extra-pulmonary malignancies.

## Case 1

A 32-year-old man presented to the Emergency Department (ED) of Hospital Regional Lambayeque in Peru on July 21, 2019. He had been diagnosed with diabetes mellitus (type 2) two years before, but he did not follow treatment because of financial reasons. He denied risky sexual behavior, smoking, alcoholism, or any previous infectious disease. Furthermore, he denied weight loss, cough, hemoptysis, sweating, diarrhea or constipation. Thirty days before admission, he reported mild colicky hypogastric pain, without irradiation. One week before admission, abdominal pain turned to severe, diffuse, and persistent; and anorexia, general malaise, and premature abdominal fullness after meals were added. Three days before admission, fever and vomiting started.

At admission, remarkable basic hematology and biochemistry were leukocytosis (15 200/μL), lymphopenia (608/μL), hypoalbuminemia (2.9 g/dL), and a glycated hemoglobin of 6.8 mg/dL. Serologies for human immunodeficiency virus (HIV), human T-lymphotropic virus type 1 (HTLV-1), hepatitis B and C, and rapid plasma reagin/Venereal Disease Research Laboratory (RPR/VDRL), were negative. A chest x-ray (CxR) was normal. An abdominal ultrasound showed free fluid (200 ml) in the pelvic cavity associated with inflammatory signs of mesenteric fat in the right iliac fossa. Acute appendicitis was diagnosed and laparotomy was scheduled. The surgical report described: “multiple and widespread adhesions, linitis plastica, carcinomatosis, hard tumors (0.3-0.5 cm) in the peritoneum, short bowels, and cecum, and multiple abscess in both iliac fossae with purulent and foul-smelling secretion” *.*

**Figure 1.**
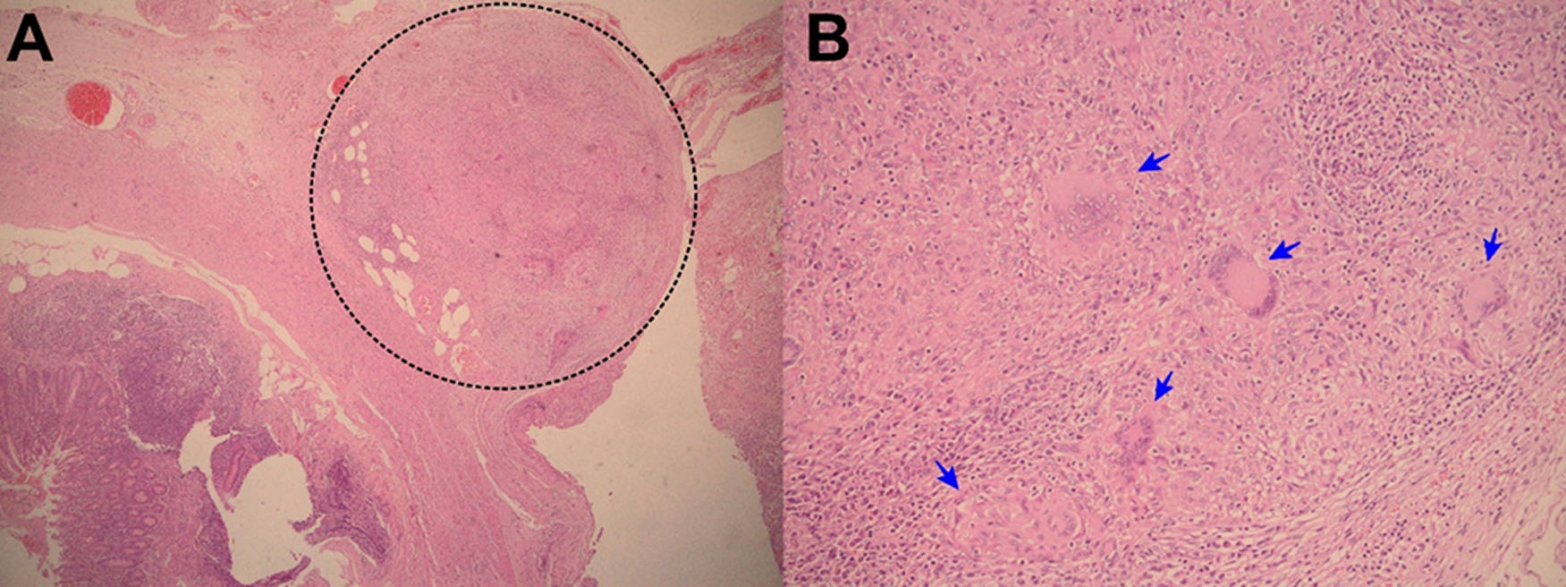
A: Electronic microphotography (x40) showing chronic granulomatous mesoappendicitis and a pseudotumoral lesion which corresponds to granulomas (circle in dotted line). B: Electronic microphotography (x40) showing Langhans multinucleated giant cells inside the granuloma (blue arrows). No caseous necrosis was seen. Ziehl-Neelsen smear for Acid Fast Bacilli was negative.

**Figure 2.**
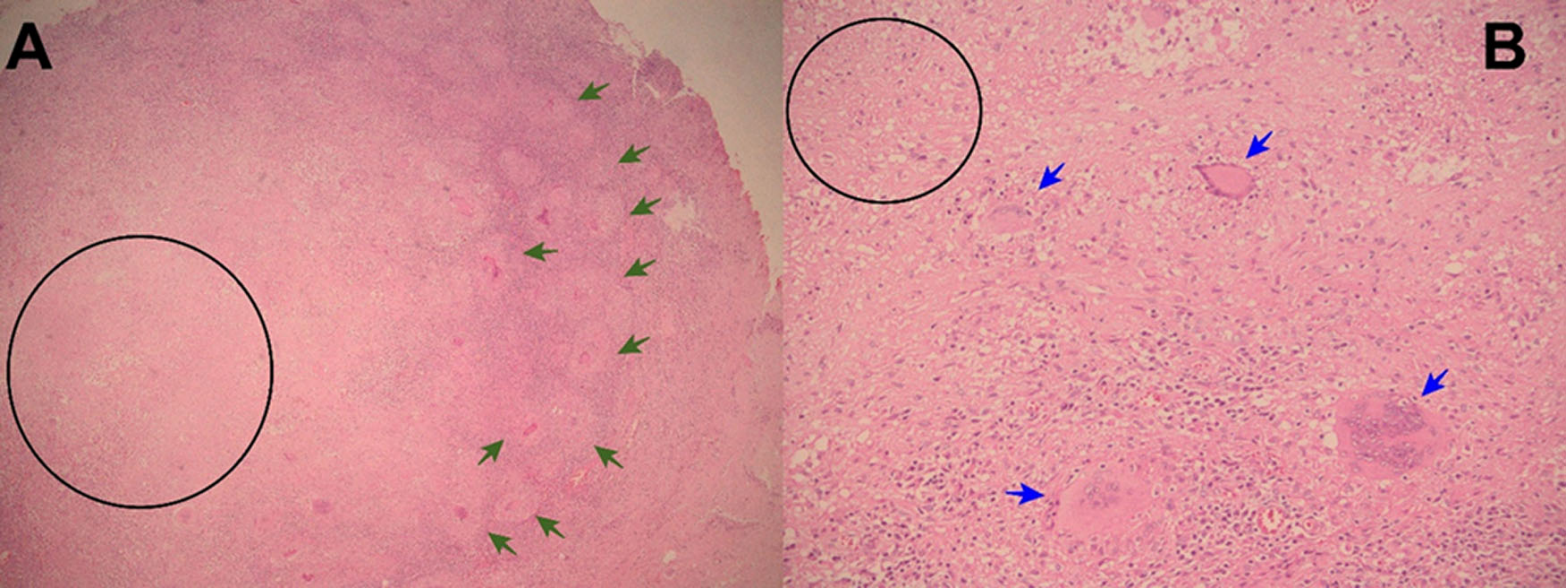
A: Electronic microphotography (x40) showing parietal peritoneum with chronic granulomatous inflammation, granulomas (green arrows), and caseous necrosis (black circle). B: Electronic microphotography (x40) showing Langhans multinucleated giant cells (blue arrows); caseous necrosis (black circle). Ziehl-Neelsen smear for Acid Fast Bacilli was negative.

**Figure 3.**
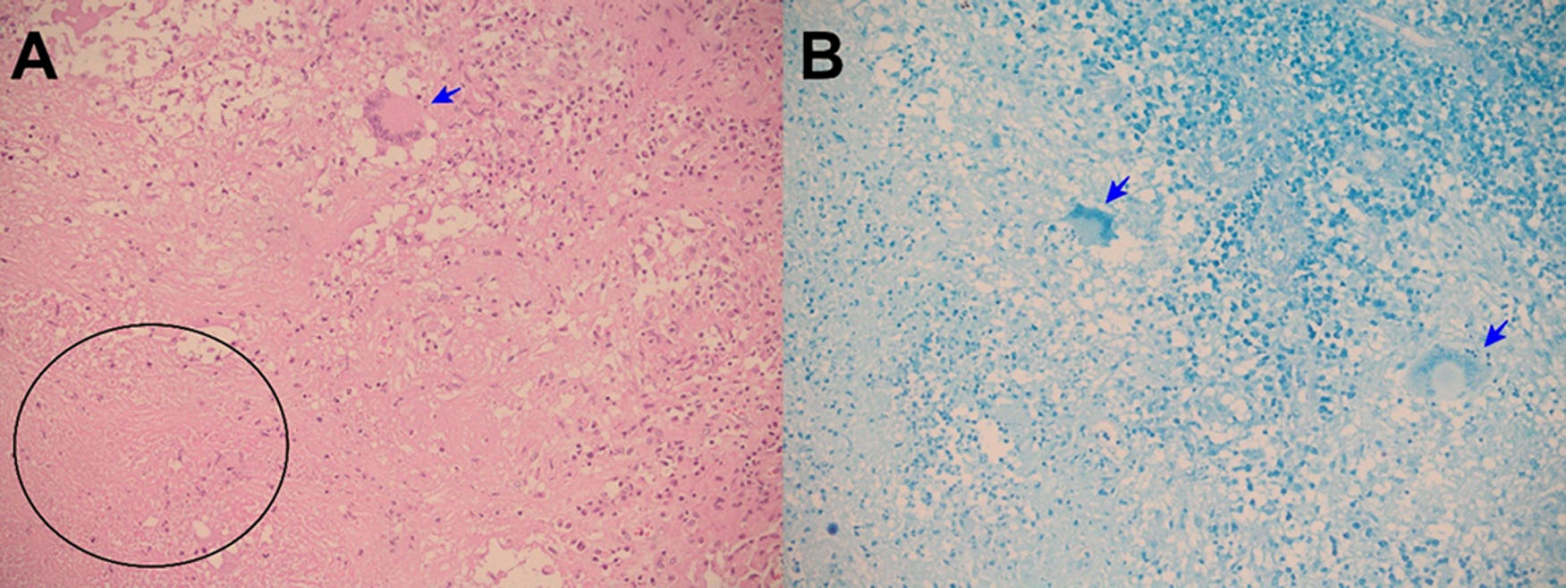
A: Electronic microphotography (x40) showing small bowel with chronic granulomatous inflammation, caseous necrosis (black circle) and Langhans multinucleated giant cells (blue arrows). B: Electronic microphotography (x40) showing Langhans multinucleated giant cells (blue arrows); Ziehl-Neelsen smear for Acid Fast Bacilli was negative.

**Figure 4.**
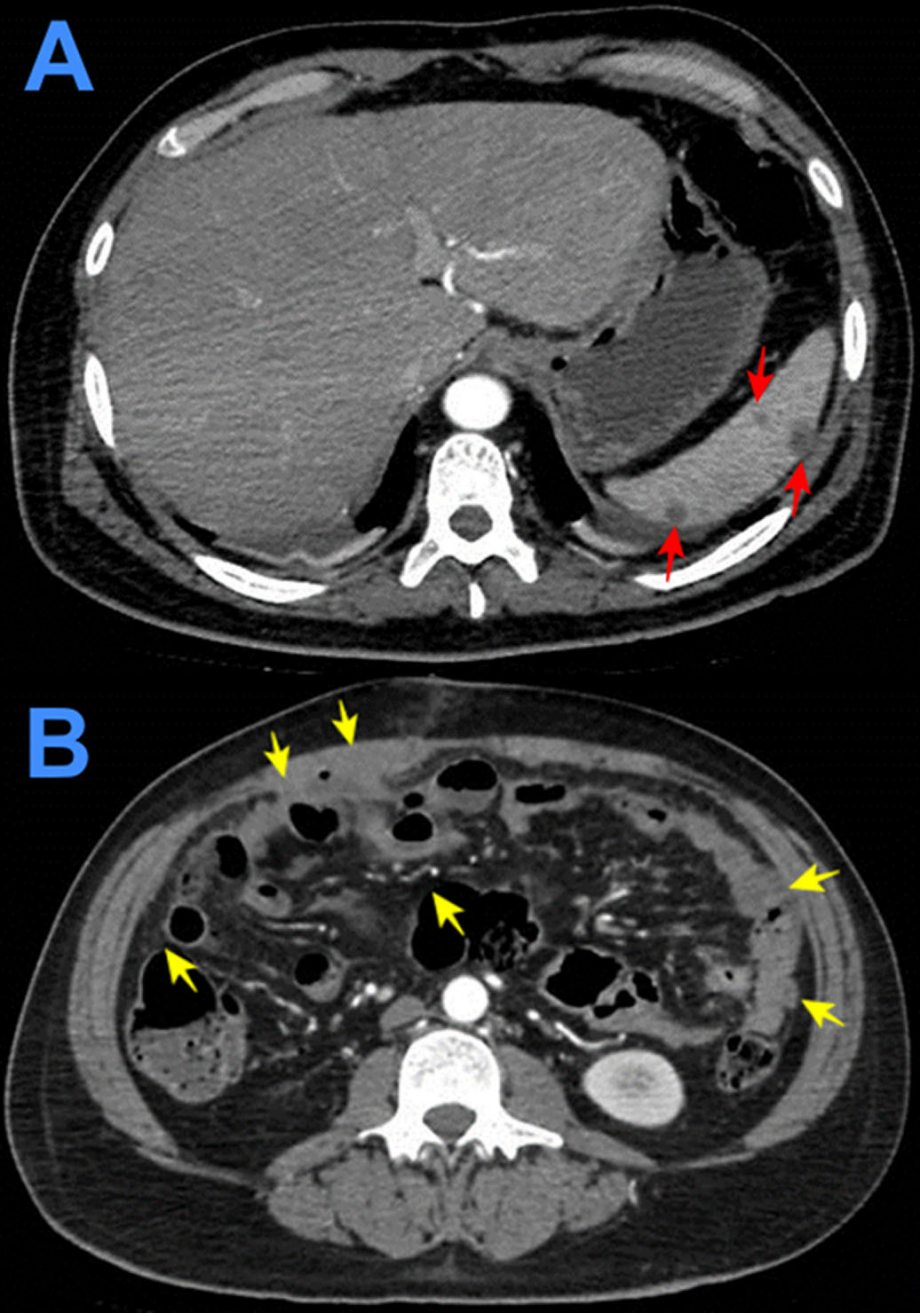
Contrast enhanced computed tomography showing solid focal hypodense areas of <20 mm in diameter that do not enhance with contrast (A). Diffuse thickening of parietal peritoneum with multiple hypodense nodular implants with well-defined borders; inflammatory changes in both iliac fosses; and scarce free fluid in the paracolic gutters and paravesical spaces (B).

*Evolution.* One hour after surgery, because of clinical signs of hemodynamic shock, the patient was transferred to the intensive care unit (ICU). At ICU admission the patient was febrile (38.5°C), with hypotension (arterial blood pressure 70/30 mmHg), tachycardia (116 beats per minute), tachypnea (26 breathes per minute) without hypoxia (peripheric saturation 96% in ambient-air), severe dehydration with cold and mottled skin with prolonged capillary refill time, with sleepiness but respondent to verbal stimulus; the abdomen was distended and painful, with reduced intestinal peristalsis, and the wound (8 cm in length) of the recent surgery was macroscopically in order and percutaneous drainages in both iliac fossa were in place showing serohematic secretion. A broad-spectrum antibiotic therapy (meropenem and metronidazole) was started, together with supportive treatment including vasopressors (noradrenaline) and fluid replacement. After a week of the above-mentioned treatment, the clinical condition of the patient was improved and at the seventh day after the surgery, the patient was transferred from the ICU to another hospital division. Then, the result of the histopathological exams of samples collected during surgery showed: “chronic granulomatous inflammation, granulomas, extensive caseous necrosis and Langhans giant multinucleated cells; Ziehl-Neelsen (ZN) stain for Acid Fast Bacilli (AFB) negative”, and a diagnosis of abdominal TB was performed ( Figures 1- 3). Based on this result quadruple anti-tuberculous treatment (isoniazid 5 mg/kg, rifampicin 10 mg/kg, ethambutol 20 mg/kg, pyrazinamide 25 mg/kg) was initiated in concordance with Peruvian and international guidelines. ^11, 12^ Subsequently, the diagnosis was confirmed by a polymerase-chain reaction (PCR) for *Mycobacterium tuberculosis* in peritoneal fluid *.* The tuberculin skin test (Mantoux or PPD test) was negative. Serial ZN smears for AFB in urine, sputum, secretion of both pelvic drains, were negative; adenosine deaminase test in peritoneal fluid 292.7 U/L (reference value < 40 U/L). Blood cultures, and cultures for common pathogens, fungi, and *M. tuberculosis* in urine and peritoneal fluid were negative. Before discharge, the patient was evaluated by an oncologist who suggested some other exams: serum tumor markers (CEA, CA 125, AFP, CA19.9, β-HCG), esophagogastroduodenoscopy, and colonoscopy with ileoscopy, all of which were within normal ranges. In addition, contrast-enhanced chest-abdomen-pelvis computed tomography (CT) and magnetic resonance imaging (MRI) showed “multiple nodular implants in spleen, gut, and peritoneal cavity; but no hepatosplenomegaly or abnormal lymph nodes enlargement” ( Figure 4). One week after the start of the antituberculosis treatment, the patient was discharged from hospital because of clinical improvement.

## Case 2

A 20-year-old man presented to the ED of Hospital Regional Lambayeque on July 18, 2020. He denied any previous disease, but had been incarcerated for almost two years before admission. He presented for six months before admission with fever, productive cough, abdominal pain, nausea and weight loss. One week before admission, he initiated with colicky abdominal pain, nausea, vomiting, and anorexia for three days. One day before admission, abdominal pain turned into intense and diffuse, and fever, persistent vomiting, and absence of passage of flatus and feces were added.

*Physical examination at admission.* The patient was emaciated and mildly dehydrated; blood pressure: 100/60 mmHg, respiratory rate: 20 breathes per minute, heart rate 136 beats per minute, temperature: 37.9 °C, and oxygen saturation: 95% at room air. Absent breathe sounds and dullness in both lung bases. Tachycardia. Distended and painful abdomen, bowel sounds diminished in frequency, McBurney’s and Blumberg's signs were positive. Awake and oriented patient, no focal deficit, no meningeal signs.

**Figure 5.**
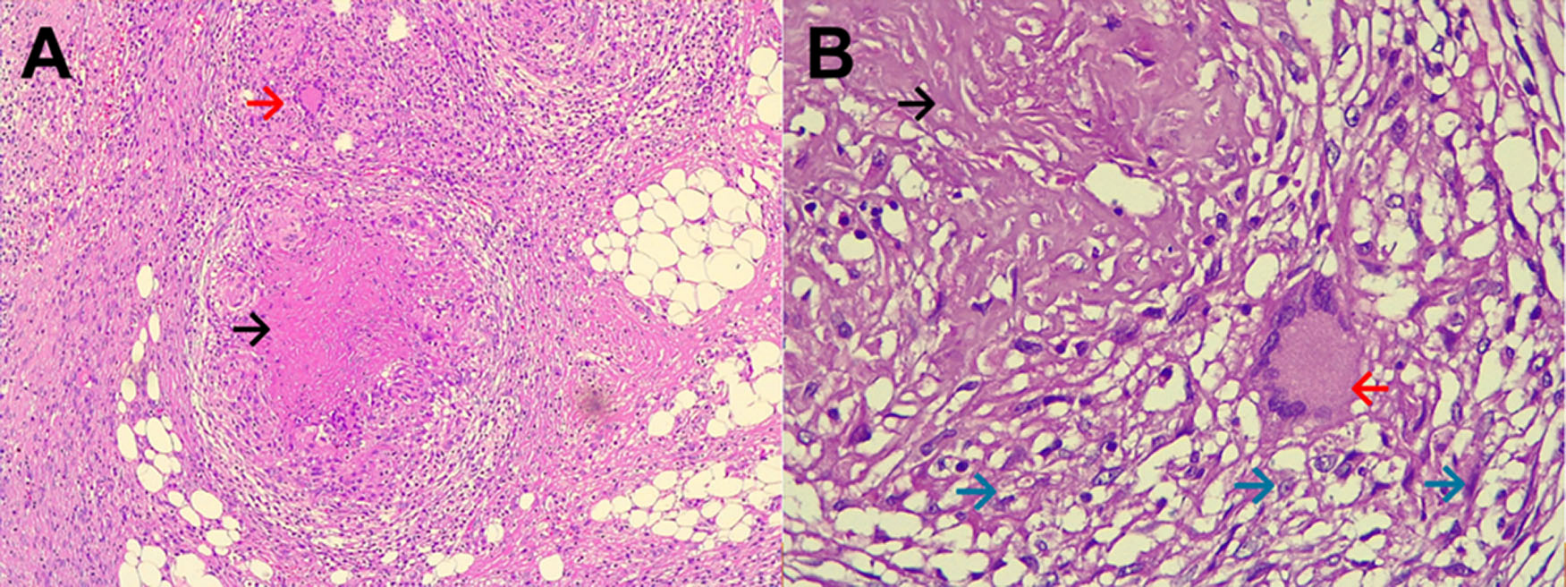
A: Electronic microphotography (x10) showing epiploon with chronic granulomatous inflammation, caseous necrosis (black arrow) and Langhans multinucleated giant cells (red arrow). B: Electronic microphotography (x40) showing Langhans multinucleated giant cells (red arrow); caseous necrosis (black arrow); and epithelioid histocytes (green arrows). Hematoxylin and Eosin stains. Periodic acid-Schiff and Ziehl-Neelsen stains were negative.

**Figure 6.**
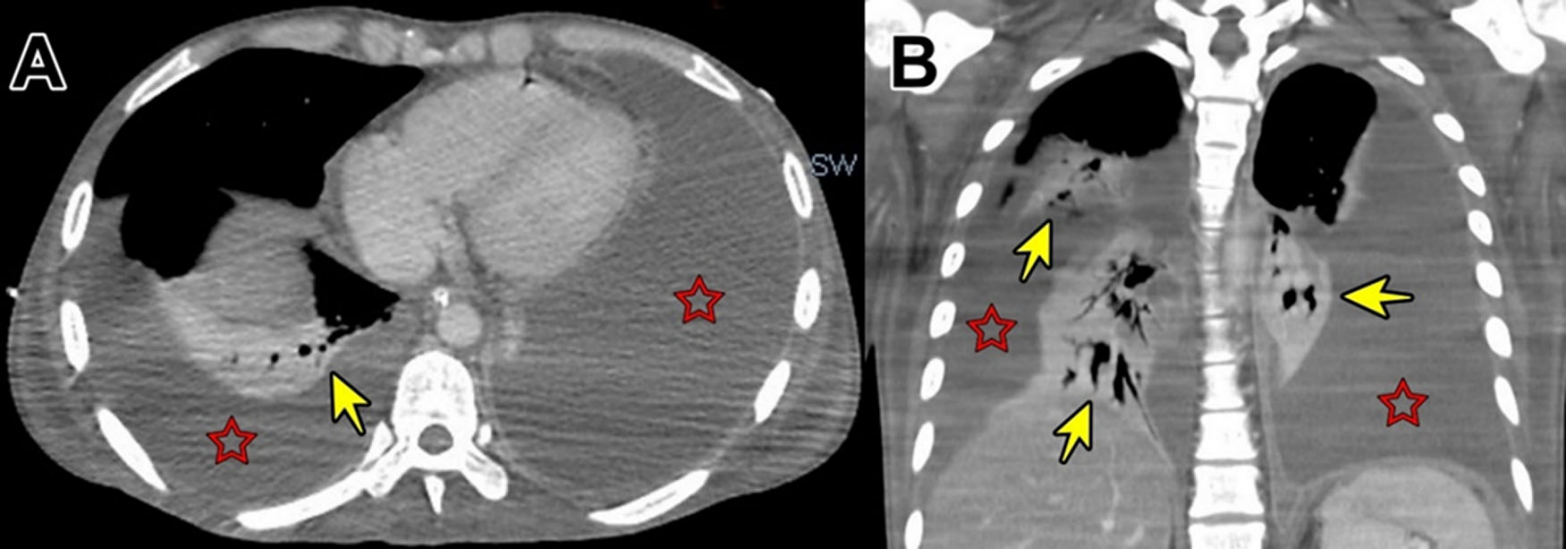
Contrast-enhanced computed tomography showing massive pleural effusion (red stars) with consolidation of both lungs (yellow arrows) (A and B).

*Evolution.* After admission, acute abdomen due to appendicitis was diagnosed. Symptomatic treatment was initiated and preoperative exams and plain chest and abdominal x-ray were ordered, which showed significant bilateral pleural effusion and ascites. Three hours after admission, the patient was transferred to the operating room. During exploratory laparotomy “multiple cerebroid nodules scattered through parietal and visceral peritoneum, small bowel, colon and epiploon; and plenty of ascitic fluid were observed”. The postoperative diagnosis was “carcinomatosis”. After surgery, he was transferred to the ICU, and continued treatment with saline, noradrenaline, imipenem, metronidazole, sedation with midazolam, analgesia with fentanyl, and ventilatory support. Based on clinical data, risk factors, intraoperative findings, and chest x ray imaging, TB was suspected. Then, stains and cultures of multiple samples for *Mycobacterium tuberculosis* (MTB) and a contrast-enhanced tomographic scan of thorax, abdomen and pelvis were ordered ( Figure 5). Three days after surgery thoracocentesis was performed. Five days after surgery RT-PCR resulted positive for MTB in pleural fluid and quadruple anti-tuberculous treatment for MTB was initiated (the same scheme as Case 1). Ten days after surgery histopathology of epiploon was obtained, showing extensive caseous necrosis with granulomas and Langhans multinucleated giant cells. The patient died seven days after initiation of anti-tuberculous treatment due to refractory septic shock ( Figure 6). A serial ZN smear for AFB in bronchial secretion, blood cultures, and urine culture, were negative. Serologies for HIV, HTLV-1, hepatitis B and C, and RPR/VDRL, were also negative.

## Discussion

Abdominal TB generally presents in four main clinical forms according to the abdominal involvement: enteritis (gastrointestinal), peritonitis, lymphadenitis, and visceral (solid organs) tuberculosis. Most of abdominal TB cases have a combination of these basic forms. ^5, 12, 13^ Tuberculous enteritis most frequently involves the ileocecal region (80-90% of cases). ^5, 13, 14^ In our patients we demonstrated enteric and peritoneal involvement.

Abdominal TB occurs by: 1) *hematogenous or lymphatic dissemination* from a primary lung focus or miliary disease; 2) *reactivation* from a latent infectious focus; 3) *contiguous spread* from mesenteric or celiac lymphatic nodes or adjacent organs (i.e. retrograde dissemination from fallopian tubes); 4) *direct mucosal invasion* by *ingestion* of tuberculous mycobacteria (ingestion of unpasteurized milk or undercooked meat). Secondary dissemination from a miliary or lung focus occurs in 10-50% of patients, and generally presents with massive involvement of the gastrointestinal tract. ^5, 13- 15^

Approximately 15-25% of patients with abdominal TB have concomitant active pulmonary TB, ^1, 13- 15^ more than 50% of patients with abdominal TB patients have some alteration in CxR suggestive of TB, ^6, 13- 15^ and one third of abdominal TB have evidence of extraperitoneal TB. ^4, 6^ This high prevalence of extrapulmonary TB suggests that abdominal TB is a part of disseminated TB. Hematogenous dissemination from a primary lung focus is probably the most important mechanism of peritoneal involvement. ^6, 15^ In our patients, we did not find active pulmonary tuberculosis.

Abdominal TB represents a diagnostic challenge and requires a high index of suspicion. Clinical manifestations of abdominal TB are variable and unspecific and can include fever, weight loss, abdominal pain and/or distension, ascites, hepatomegaly, diarrhea, intestinal obstruction, and abdominal mass. ^5, 15^ Tuberculosis can be confused with other diseases. The differential diagnosis of abdominal tuberculosis includes abdominal cancers, sarcoidosis and Crohn´s disease. These entities can imitate clinical, radiologic, endoscopic, and even laparoscopic features of entero-peritoneal tuberculosis. Thus, it is of paramount importance to take biopsies. ^5, 15- 18^ Direct biopsy of suspicious lesions by laparoscopy or laparotomy is the most certain method to obtain diagnostic samples. ^4- 6^

Histopathology (necrotizing or caseous granulomas and/or AFB) is highly suggestive and virtually diagnostic of tuberculosis, but it is not specific or pathognomonic. However, in the right clinical and epidemiological context, typical findings can support a presumptive diagnosis of TB ^1, 6, 14- 16^ as occurred in our patients. Hepatic biopsy has a sensitivity of 68% for caseating granulomas in hepatic biopsies. ^15, 18^ But, among patients with colonic tuberculosis, only 32.6% have caseous granulomas, and 5% have AFB in colonic biopsies. ^18^

A definitive diagnosis of abdominal TB is made by demonstration of *Mycobacterium tuberculosis* (in peritoneal fluid or involved organ) by culture and/or PCR. The sensitivity of AFB smear and mycobacterial culture for ascitic fluid is low (<2% and <20%, respectively); whereas, the sensitivity of AFB smear and mycobacterial culture for biopsy specimens is in general <50%. ^1, 16^ PCR is more sensitive and specific for diagnosis of TB than AFB smear or mycobacterial culture, but the diagnostic yield of PCR varies depending on the tissue type. The sensitivity and specificity are high for peritoneal fluid and pancreatic and hepatic tissue, but intestinal tissue may be associated with false-positive PCR results. ^1, 15^

Abdominal tuberculosis is the great mimicker of abdominopelvic pathology. ^4, 8- 10^ There exist a few reports of cases initially diagnosed as cancer that subsequently were proved to be tuberculosis. ^8- 10^ In a study with 170 patients referred to the National Institute of Neoplastic Diseases of Peru because of a presumed diagnosis of cancer, the final diagnosis was extrapulmonary TB in 77.7% of cases, and pulmonary TB in the 22.3% remaining. Among these 170 patients, three patients were admitted with diagnosis of gastrointestinal cancer and 17 with ovarian cancer; and entero-peritoneal TB was finally diagnosed in nine, and genitourinary TB in seven patients. Despite extrapulmonary TB accountings for up to 20-25% of TB cases in non-HIV infected patients, it is the most common localization that could mimic cancer, probably due to the fact that solid lesions such as tuberculomas —more common in not severely immunocompromised patients— tend to be more frequently confused with malignancies. Besides, in 63.5% of patients the extension of the “neoplastic” lesions would suggest an advanced-stage disease. ^8^ This interpretation could have profound implications in the management of these patients, because assuming a case as an advanced-stage cancer would reduce the chance of performing more exhaustive studies to get a definitive diagnosis. Consequently, most of these patients who are considered erroneously to have advanced cancer are offered only palliative care. Conversely, there exist reports of cancers mistakenly diagnosed and treated as tuberculosis. ^15^

Prompt diagnosis and treatment can reduce morbidity and mortality, and additionally can limit tuberculosis dissemination especially if there exist concomitant pulmonary disease. Almost all abdominal TB cases offered proper treatment result in complete remission and prognosis is excellent. Urgent surgery is only necessary in a minority of cases, if there is obstruction, ischemia, perforation, massive bleeding, or secondary peritonitis. ^4, 15, 16^

These cases are enlightening and worthy of reflection because of several reasons: 1) the rare presentation of the clinical picture as an acute surgical abdomen, septic shock, and “carcinomatosis”; 2) the absence of other causes of immunosuppression, except for diabetes in one case; 3) although, histopathology did not establish a definitive diagnosis, it did provide important diagnostic clues to support a presumptive diagnosis and initiation of empiric antituberculosis treatment, until TB was confirmed subsequently by RT-PCR.

In conclusion, these cases exemplify that we must employ our clinical judgement in conjunction with all diagnostic tools available in order to make an accurate diagnosis within the shortest possible time in patients with a clinical picture suggestive of malignancy. We must be aware that TB can mimic cancer, but the opposite is also true. In consequence, clinicians should always consider TB among the differential diagnosis of malignancy, especially in high endemicity regions or countries with high rates of immigrants **.**

## Data Availability

All data underlying the results are available as part of the article and no additional source data are required.

## Consent

**Informed Consent:** The patients provided written consent to the publication of this clinical practice article and pictures. This study was approved by the Ethics and Research Committee of Hospital Regional Lambayeque (approval number: 0321-095-20 CEI).

**Declaration of Helsinky:** The Authors have respected the Declaration of Helsinki as a statement of ethical principles for medical research involving human subjects, including research on identifiable human material and data.
