# Supplementary material for: Abdominal tuberculosis misdiagnosed as acute surgical abdomen and carcinomatosis
Source: F1000Res. 2021 Jul 12;10:355. Originally published 2021 May 7. [Version 2] doi: 10.12688/f1000research.53036.2 (PMC8243228; doi:10.12688/f1000research.53036.2)
Supplement: Peer review history

# Peer review: Reviewer response for version 1

**DOI:** 10.5256/f1000research.56377.r85111

**Published:** 2021-06-29

**Authors:** Virgilio Efrain Failoc Rojas

**Affiliations:** 1 Research Unit for the Generation and Synthesis of Evidence in Health, Universidad San Ignacio de Loyola, Lima, Peru

It is an interesting case from an academic point of view. Two cases start as acute abdominal pain due to appendicitis, resembling intraoperative carcinomatosis, and later diagnosed as tuberculosis.

 I have some suggestions: 

1. I suggest that the sentences of the evolution of the case, both the first and second, should be structured in two or three paragraphs.
2. In the discussion I suggest highlighting a little more the fact that tuberculosis is the great imitator and in such an endemic country it should be sought in patients that in the face of a bad evolution this pathology should be sought, as it could be economically beneficial (Foster *et al.*, 2021 ^1^). 

Are enough details provided of any physical examination and diagnostic tests, treatment given and outcomes?

Yes

Is the background of the cases’ history and progression described in sufficient detail?

Yes

Is sufficient discussion included of the importance of the findings and their relevance to future understanding of disease processes, diagnosis or treatment?

Yes

Is the conclusion balanced and justified on the basis of the findings?

Yes

Reviewer Expertise:

Medicine clinical and research

I confirm that I have read this submission and believe that I have an appropriate level of expertise to confirm that it is of an acceptable scientific standard.

## References

[1] : Strengthening health systems to improve the value of tuberculosis diagnostics in South Africa: A cost and cost-effectiveness analysis. *PLoS One* .2021;16(5) : 10.1371/journal.pone.0251547 e0251547 10.1371/journal.pone.0251547 33989317PMC8121360

# Peer review: Reviewer response for version 1

**DOI:** 10.5256/f1000research.56377.r85112

**Published:** 2021-06-01

**Authors:** Uzair Yaqoob

**Affiliations:** 1 Dow University of Health Sciences, Karachi, Pakistan

**General comments:**

 The authors describe two cases of abdominal tuberculosis, initially presenting as acute abdomen due to appendicitis, resembling carcinomatosis intraoperatively, and later diagnosed as tuberculosis on investigations. I have a few suggestions for revision:

**Specific comments:**

Abstract:

1. I do not think "intra-operatory" is the correct word.
2. Abstract is starting with tuberculosis in general and there is no transition to abdominal tuberculosis.

Introduction:

1. The following sentence should be split into 2 or 3 sentences "At ICU admission the patient was febrile (38.5°C), with hypotension (arterial blood pressure 70/30 mmHg), tachycardia (116 beats per minute), tachypnea (26 breathes per minute) without hypoxia (peripheric saturation 96% in ambient-air), severe dehydration with cold and mottled skin with prolonged capillary refill time, with sleepiness but respondent to verbal stimulus; the abdomen was distended and painful, with reduced intestinal peristalsis, and the wound (8 cm in length) of the recent surgery was macroscopically in order and percutaneous drainages in both iliac fossa were in place showing serohematic secretion."
2. "diagnosis of abdominal TB was performed" - I do not think 'performed' is the correct verb here.

Discussion:

1. Needs more of the text relating literature with the two presented cases.

Are enough details provided of any physical examination and diagnostic tests, treatment given and outcomes?

Yes

Is the background of the cases’ history and progression described in sufficient detail?

Yes

Is sufficient discussion included of the importance of the findings and their relevance to future understanding of disease processes, diagnosis or treatment?

Yes

Is the conclusion balanced and justified on the basis of the findings?

Yes

Reviewer Expertise:

Surgical and public health research.

I confirm that I have read this submission and believe that I have an appropriate level of expertise to confirm that it is of an acceptable scientific standard.
